# Antihypertensive Effects of a Sodium Thiosulfate-Loaded Nanoparticle in a Juvenile Chronic Kidney Disease Rat Model

**DOI:** 10.3390/antiox13121574

**Published:** 2024-12-20

**Authors:** You-Lin Tain, Chien-Ning Hsu, Chih-Yao Hou, Chih-Kuang Chen

**Affiliations:** 1 Department of Pediatrics, Kaohsiung Chang Gung Memorial Hospital, Kaohsiung 833, Taiwan; tainyl@cgmh.org.tw; 2 College of Medicine, Chang Gung University, Taoyuan 330, Taiwan; 3 Institute for Translational Research in Biomedicine, Kaohsiung Chang Gung Memorial Hospital, Kaohsiung 833, Taiwan; 4 Department of Pharmacy, Kaohsiung Chang Gung Memorial Hospital, Kaohsiung 833, Taiwan; cnhsu@cgmh.org.tw; 5 School of Pharmacy, Kaohsiung Medical University, Kaohsiung 807, Taiwan; 6 Department of Seafood Science, National Kaohsiung University of Science and Technology, Kaohsiung 811, Taiwan; chihyaohou@webmail.nkmu.edu.tw; 7 Polymeric Biomaterials Laboratory, Department of Materials and Optoelectronic Science, National Sun Yat-Sen University, Kaohsiung 804, Taiwan

**Keywords:** chronic kidney disease, hydrogen sulfide, hypertension, nitric oxide, renin–angiotensin system, sodium thiosulfate, nanoparticles

## Abstract

Sodium thiosulfate (STS), a precursor of hydrogen sulfide (H_2_S), has demonstrated antihypertensive properties. Previous studies have suggested that H_2_S-based interventions can prevent hypertension in pediatric chronic kidney disease (CKD). However, the clinical application of STS is limited by its rapid release and intravenous administration. To address this, we developed a poly-lactic acid (PLA)-based nanoparticle system for sustained STS delivery and investigated whether weekly treatment with STS-loaded nanoparticles (NPs) could protect against hypertension in a juvenile CKD rat model. Male Sprague Dawley rats, aged three weeks, were fed a diet containing 0.5% adenine for three weeks to induce a model of pediatric CKD. STS-loaded NPs (25 mg/kg) were administered intravenously during weeks 6, 7, and 8, and at week 9, all rats were sacrificed. Treatment with STS-loaded NPs reduced systolic and diastolic blood pressure by 10 mm Hg and 8 mm Hg, respectively, in juvenile CKD rats. The protective effect of STS-loaded NPs was linked to increased renal expression of H_2_S-producing enzymes, including cystathionine γ-lyase (*CSE*) and D-amino acid oxidase (*DAO*). Additionally, STS-loaded NP therapy restored nitric oxide (NO) signaling, increasing L-arginine levels, which were disrupted in CKD. Furthermore, the beneficial effects of STS-loaded NPs were associated with inhibition of the renin–angiotensin system (RAS) and the enhancement of the NO signaling pathway. Our findings suggest that STS-loaded NP treatment provides sustained STS delivery and effectively reduces hypertension in a juvenile CKD rat model, bringing us closer to the clinical translation of STS-based therapy for pediatric CKD-induced hypertension.

## 1. Introduction

Hydrogen sulfide plays a variety of biological roles, including antioxidant, anti-inflammatory, anti-apoptotic, and vasodilatory effects, as well as contributing to mitochondrial bioenergetics [1]. H_2_S plays a role in regulating blood pressure (BP), and reduced levels have been linked to the development of chronic kidney disease (CKD) and hypertension [1,2,3]. CKD and hypertension are closely linked in a bidirectional relationship, where one condition can exacerbate the other. Hypertension can both initiate and accelerate the progression of CKD. Hypertension is one of the most common comorbidities in patients with CKD. Given this strong connection, managing high BP is essential in individuals with CKD to slow kidney damage and break the vicious cycle.

H_2_S is produced through three pathways: enzymatic, non-enzymatic, and bacterial. Endogenously, it is generated from L-cysteine by the enzymes cystathionine β-synthase (*CBS*), cystathionine γ-lyase (*CSE*), 3-mercaptopyruvate sulfurtransferase (*3MST*), and D-amino acid oxidase (*DAO*) [1]. Non-enzymatic production occurs via sulfan sulfur, with thiosulfate, a major oxidation product of H_2_S, being reducible back to H_2_S. Clinically, sodium thiosulfate (STS), a form of thiosulfate, is used to treat conditions like calciphylaxis, cyanide toxicity, carbon monoxide poisoning, and cisplatin-induced damage [4]. Beyond being an H_2_S donor, STS also has antioxidant and anti-inflammatory effects, making it a promising therapeutic candidate [4].

In children, CKD is a leading cause of hypertension [5], often resistant to multiple antihypertensive treatments [6]. As CKD is a major contributor to resistant hypertension [7], the search for novel treatments, particularly for children, warrants greater attention. The addition of adenine to the diet of rats induces kidney injury, serving as a model that mimics human CKD [8]. Our previous study showed that juvenile rats fed a diet supplemented with 0.5% adenine for 3 weeks experienced a decline in kidney function and hypertension, resembling pediatric CKD [9]. In this model, oral administration of STS for two weeks reduced systolic and diastolic BP by 7 and 9 mm Hg, respectively [10].

Nanotechnology has made significant contributions to medicine, particularly in the targeted delivery of drugs to specific organs, such as the kidneys [11]. Recently, extensive research has focused on developing novel nanoplatforms for H_2_S-based therapies, aiming to improve the precision and effectiveness of treatments related to H_2_S [12].

Using the mini-emulsion interfacial crosslinking technique, we have developed polylactide (PLA)-based biodegradable nanoparticles (NPs) as drug delivery carriers [13]. Our goal was to further develop an STS-loaded NP system for sustained STS delivery and evaluate its effectiveness in a juvenile rat model of CKD.

## 2. Materials and Methods

### 2.1. Preparation of STS-Loaded NPs

We previously synthesized the poly (ethylene glycol)-block-polylactide-block-allyl functional polylactide (PEG-PLA-APLA) precursor [13]. The unsaturated double bonds on the APLA segments were then modified through a thiol-ene click reaction with 2-(Diethylamino)ethanethiol hydrochloride (DEAET), converting the APLA segments into cationic PLA (CPLA), which imparted positive charges to the polymer. The resulting PEG-PLA-CPLA was used as a polymeric surfactant, with Pluronic^®^ F-127 (F127) added as a non-ionic macromolecular surfactant. The inclusion of F127 enhanced the interaction with the synthesized polymers, improving interfacial adsorption and colloidal dispersion stability [14].

First, 10 mg of PEG-PLA-CPLA and F127 were dissolved in 2 mL of dichloromethane (DCM), and the PEG-PLA-CPLA/F127 solution was combined with 0.3 mL of deionized water in a centrifuge tube. The mixture was subjected to ultrasonic emulsification using a sonicator (Q700, Qsonica, Newtown, CT, USA) for 1 min to form a primary water-in-oil (*w*/*o*) emulsion. To assess the effects of F127 concentration (5, 10, and 20 mg) and the volume of deionized water (0.3, 0.4, and 0.5 mL) on NP size and morphology, these parameters were varied during the process. The primary emulsion was then mixed with 4 mL of a 2% (*w*/*v*) polyvinyl alcohol (PVA) solution and sonicated for an additional 2 min to create a double emulsion (w/o/w). This double emulsion was slowly added dropwise to 6 mL of a 0.6% (*w*/*v*) PVA solution and stirred on a magnetic stirrer for 6 h to allow for the complete evaporation of DCM. Finally, the PEG-PLA-CPLA/F127 NPs were collected by centrifugation at 27,670× *g*-force for 20 min at room temperature and washed three times with ultrapure water.

For the preparation of STS-loaded NPs, the procedure was the same as for the PEG-PLA-CPLA/F127 NPs, with the exception that 5 mg of STS was pre-dissolved in the deionized water phase (0.3, 0.4, or 0.5 mL) used for the primary emulsion. After the additional ultrasonic emulsification, solvent removal, and purification steps, the STS-loaded NPs were obtained. To evaluate the drug loading capacity, the supernatant from the first centrifugation step was collected and analyzed to determine the amount of unencapsulated STS using a UV–vis spectrophotometer (V-770, Jasco Inc., Tokyo, Japan) at a wavelength of 250 nm, based on a standard calibration curve of STS in water. The drug loading content (LC) and encapsulation efficiency (EE) of the STS-loaded NPs were calculated using the following equations:

LC (wt%) = (weight of loaded STS/weight of NP) × 100%(1)

EE (wt%) = (weight of loaded STS/weight of initial STS feeding) × 100%(2)

### 2.2. Characterization of NPs

The nanoparticle size (mean diameter, Dav) and polydispersity index (PDI) were analyzed using dynamic light scattering (DLS, Nanotrac Wave II, Microtrac). The viscosity and refractive index parameters for the tests were set according to those of aqueous solutions, with each sample tested in triplicate. The colloidal stability of the NP samples was assessed in phosphate-buffered saline (PBS) at pH 7.4 and 5.5, as well as in a 2 M NaCl dispersion medium. The NP samples were incubated at 37 °C, with measurements taken at time points of 1, 3, 5, and 7 days using DLS. The NP concentration was maintained at 1 mg/mL throughout the experiment.

The shape and morphology of both PEG-PLA-CPLA/F127 NPs and STS-loaded NPs were examined by transmission electron microscopy (TEM, JEOL Ltd., Akishima, Japan). For TEM imaging, 10 μL of a 0.1 mg/mL NP solution was placed onto a 300-mesh carbon-coated copper grid, stained with ruthenium tetroxide, air-dried at room temperature for 12 h, and then placed under vacuum for 24 h. TEM images were acquired using a TEM (JEM-2100, JEOL Ltd., Akishima, Japan).

### 2.3. In Vitro STS Release Studies

To test the in vitro release of STS from the NPs, STS-loaded NPs (containing 1 mg of STS) were redispersed in PBS buffer solutions (pH = 7.4 and 5.5, respectively). The solution was then sealed in a dialysis bag (MWCO: 3 kDa) and placed in the same buffer medium. Stirring was conducted using a magnetic stirrer at 100 r.p.m., with the temperature maintained at 37 °C. At predetermined time points, 3 mL of the medium was withdrawn for analysis, and an equal volume of fresh PBS buffer was added to maintain the volume. The STS concentration was determined using a UV spectrophotometer (V-770, Jasco Inc., Tokyo, Japan) at a wavelength of 250 nm, based on a previously established STS standard calibration curve. Drug release experiments were performed in triplicate for each time point, and the mean values were calculated.

### 2.4. Animals

Male 3-week-old Sprague Dawley (SD) rats (50–55 g, *n* = 32) were obtained from BioLASCO Taiwan Co., Ltd. (Taipei, Taiwan). Upon arrival, the rats were allowed to acclimate in a temperature-controlled room (22 ± 1 °C), with 55 ± 5% humidity and a 12:12 light–dark cycle, in an AAALAC International-accredited animal facility. Two rats were housed per cage. Rats were given unrestricted access to standard chow (Altromin 1324, Altromin Spezialfutter GmbH & Co. KG, Lage, Germany) and tap water. All procedures were performed in accordance with the NIH Guide for the Care and Use of Laboratory Animals and approved by the Institutional Animal Care and Use Committee (IACUC) of Chang Gung Memorial Hospital (Permit #2023081102; approval date: 28 September 2023). The rats were randomly assigned to one of four groups (*n* = 8 per group): normal control (C), fed regular chow; CKD group, fed a diet supplemented with 0.5% adenine from 3 weeks of age for 3 weeks; regular chow with STS-loaded NPs; and CKD with STS-loaded NPs. PEG-PLA-CPLA/F127-based STS-releasing NPs were administered via tail vein injection at a dose of 25 mg/kg weekly during weeks 6, 7, and 8. The dose of NPs used here was based on previous studies [15].

BP was measured using the CODA rat tail-cuff system (Kent Scientific Corporation, Torrington, CT, USA) [7]. All rats underwent a 1-week acclimation period to restraint and tail-cuff inflation to ensure accurate and reproducible measurements. The rats were sacrificed at 9 weeks of age. The rats were initially anesthetized with an intraperitoneal injection of ketamine (50 mg/kg) and xylazine (10 mg/kg). Following anesthesia, they were humanely euthanized via an intraperitoneal overdose of pentobarbital. Blood samples were collected into heparinized tubes, and kidneys were immediately snap-frozen and stored at −80 °C for subsequent analysis.

### 2.5. Quantitative PCR

Total RNA was extracted from kidney cortex samples, and real-time quantitative PCR (qPCR) was performed as previously described in our methods [7]. Briefly, complementary DNA (cDNA) was synthesized using MMLV Reverse Transcriptase (Invitrogen, Carlsbad, CA, USA). Two-step quantitative real-time PCR was performed with the Quantitect SYBR Green PCR Reagents kit (Qiagen, Valencia, CA, USA) and the iCycler iQ Real-Time PCR detection system (Bio-Rad, Hercules, CA, USA). The cycling conditions included one cycle of 3 min denaturation at 95 °C, followed by 45 amplification cycles (95 °C for 10 s, 55–60 °C (gene-dependent) for 20 s, 72 °C for 1 s), and a three-segment melting cycle (95 °C for 5 s, 65 °C for 30 s, 97 °C for 5 min).

The expression levels of H_2_S-producing enzymes and RAS components were analyzed. All samples were run in duplicate, with 18S ribosomal RNA (*R18S*) used as the reference gene for data normalization. The primer sequences are listed in Table 1. For relative quantification, the comparative threshold cycle (CT) method was applied. The average CT was subtracted from the corresponding averaged *R18S* value to obtain ∆CT, and ∆∆CT was calculated by subtracting the control ∆CT from the experimental ∆CT. The fold change was calculated as 2^−∆∆CT^.

### 2.6. High-Performance Liquid Chromatography (HPLC)

Several biochemical parameters of the nitric oxide (NO) pathway, including L-citrulline, L-arginine, symmetric dimethylarginine (SDMA), and asymmetric dimethylarginine (ADMA), were measured using an Agilent 1100 HPLC system (Santa Clara, CA, USA) with the OPA-3MPA derivatization reagent [10]. The plasma concentrations of L-citrulline and L-arginine, which serve as substrates for NO production, were measured in duplicate. Additionally, the plasma levels of the NO synthase inhibitors SDMA and ADMA were also assessed. The L-arginine-to-ADMA ratio was calculated to assess NO bioavailability [16]. Plasma creatinine levels were measured by HPLC, following a protocol validated in our laboratory.

### 2.7. Statistics

Quantitative data are expressed as the mean ± standard error of the mean (SEM). Statistical analyses were performed using one- or two-way ANOVA. A *p*-value of less than 0.05 was considered statistically significant, and Tukey’s post hoc test was applied for pairwise comparisons when appropriate. Statistical analysis was conducted using the SPSS software 22.0 (SPSS Inc., Chicago, IL, USA).

## 3. Results

### 3.1. Characterization of STS-Loaded NPs

**Figure 1:**
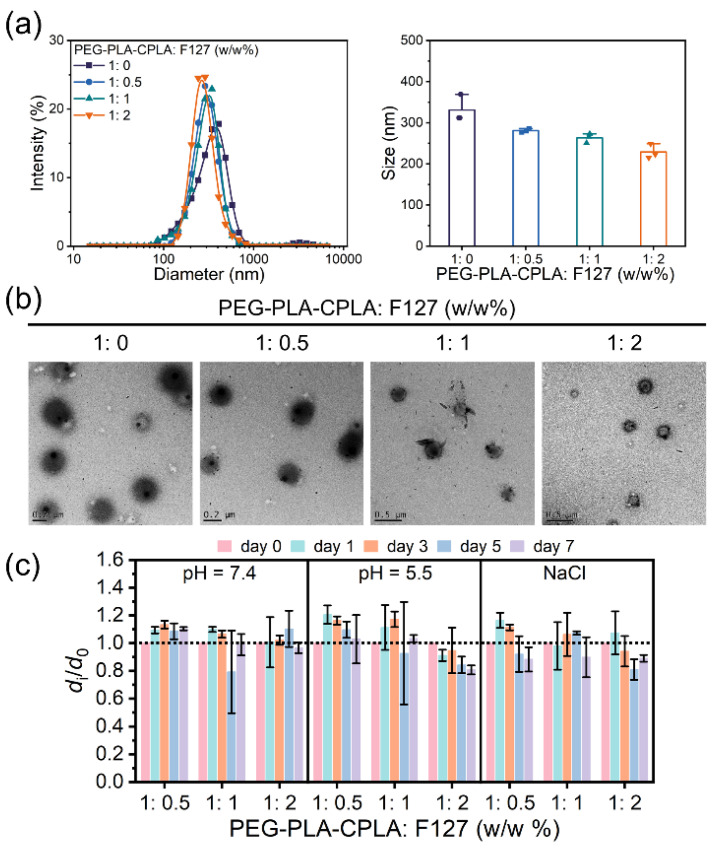
(**a**) Size distribution and average size of PEG-PLA-CPLA/F127 NPs prepared with different weight ratios of PEG-PLA-CPLA to F127 (1:0.5, 1:1, and 1:2); (**b**) TEM images of PEG-PLA-CPLA/F127 NPs. (**c**) *d*_i_/*d*_0_ values of PEG-PLA-CPLA/F127 NPs in PBS buffer at pH = 7.4 and 5.5, and NaCl, as a function of increased incubation time.

We first investigated the influence of varying ratios of PEG-PLA-CPLA and F127 on the characteristics of the PEG-PLA-CPLA/F127 NPs (Figure 1). The particle size results showed that PEG-PLA-CPLA NPs had a size of 331 nm, and a significant reduction in particle size was observed as the F127 proportion increased (Figure 1a). Specifically, as the PEG-PLA-CPLA-to-F127 ratio increased from 1:0.5 (*w*/*w*%) to 1:2 (*w*/*w*%), the particle size decreased from 281 nm to 229 nm, with a uniform size distribution. TEM images revealed that the PEG-PLA-CPLA/F127 NPs exhibited a well-defined, smooth, spherical structure with consistent size and shape (Figure 1b). Notably, F127 was found to reduce the particle size without altering the spherical morphology, confirming the successful preparation of NPs via the double-emulsion method.

To assess the NPs’ stability under simulated physiological conditions, we incubated them in PBS buffers at pH 7.4 and 5.5, as well as in an NaCl medium, at 37 °C. Particle size changes were monitored, and the di/d_0_ ratio was used to quantify the degree of change (Figure 1c), where di represents the Dav after i days of incubation, and d_0_ is the initial Dav measured immediately after NP preparation. The results indicated that NPs prepared with a PEG-PLA-CPLA-to-F127 ratio of 1:0.5 (*w*/*w*%) maintained a di/d_0_ ratio of 1:1 after 7 days of incubation in the selected medium, suggesting that the NPs retained their original size without significant aggregation or disintegration. This demonstrated enhanced colloidal stability, allowing the NPs to maintain structural integrity in physiological environments. In contrast, NPs prepared with PEG-PLA-CPLA-to-F127 ratios of 1:1 (*w*/*w*%) and 1:2 (*w*/*w*%) exhibited greater fluctuations in the di/d_0_ values. Since F127 is a thermally responsive polymer, we hypothesize that an excess of F127 at 37 °C may affect the original PEG-PLA-CPLA NP structure over time. Consequently, the NPs prepared with a PEG-PLA-CPLA-to-F127 ratio of 1:0.5 (*w*/*w*%) were selected for further study in this research.

**Figure 2:**
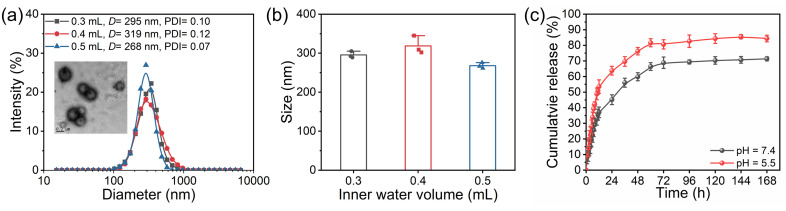
Size distribution of STS-loaded NPs and average particle size (**a**) during the first emulsion stage under varying aqueous-phase volumes; and (**b**) drug release profiles of STS-loaded NPs in PBS buffer at pH 7.4 and 5.5. (**c**) In the formulation of STS-loaded NPs, the ratio of PEG-PLA-CPLA to F127 was 1:0.5 (*w*/*w*%), with a fixed STS mass of 5 mg.

After formulating the PEG-PLA-CPLA/F127 NPs, we investigated the impact of varying the aqueous-phase volume used during the initial emulsion step on both the encapsulation efficiency of STS and the overall NP characteristics, using a fixed STS dosage of 5 mg. The particle size of the STS-loaded NPs was measured by dynamic light scattering (DLS) following the encapsulation process (Figure 2a,b). At an aqueous-phase volume of 0.3 mL, the mean particle diameter (Dav) was 295 nm; with 0.4 mL, the Dav increased to 319 nm; and at 0.5 mL, the Dav decreased to 268 nm.

To evaluate the efficiency of STS encapsulation, UV–vis spectrophotometry was employed, revealing loading efficiencies between 78 wt% and 79.2 wt% as the aqueous-phase volume increased from 0.3 mL to 0.5 mL. Based on these results—taking into account both particle size and STS loading efficiency—we selected 0.5 mL as the optimal aqueous-phase volume for the NP formulation. This volume offered a balanced compromise, achieving efficient drug encapsulation while maintaining desirable nanoparticle characteristics, such as size and stability.

We next investigated the release behavior of STS-loaded NPs under conditions of 37 °C in PBS buffer at pH 7.4 and pH 5.5 (Figure 2c). The results showed that at pH 7.4, STS-loaded NPs released STS in a slow and sustained manner, with 45.2% of the drug released over 24 h. In contrast, at pH 5.5, the release rate was faster, with 63.8% of STS released within the same time frame. After 72 h, the cumulative release was 68.8% at pH 7.4 and 80.6% at pH 5.5. As shown in Figure 1c, PEG-PLA-CPLA/F127 NPs exhibited instability under acidic conditions (pH 5.5), which contributed to the faster release of STS compared to the release at pH 7.4.

### 3.2. Effects of STS-Loaded NPs on Juvenile CKD Rats

**Figure 3:**
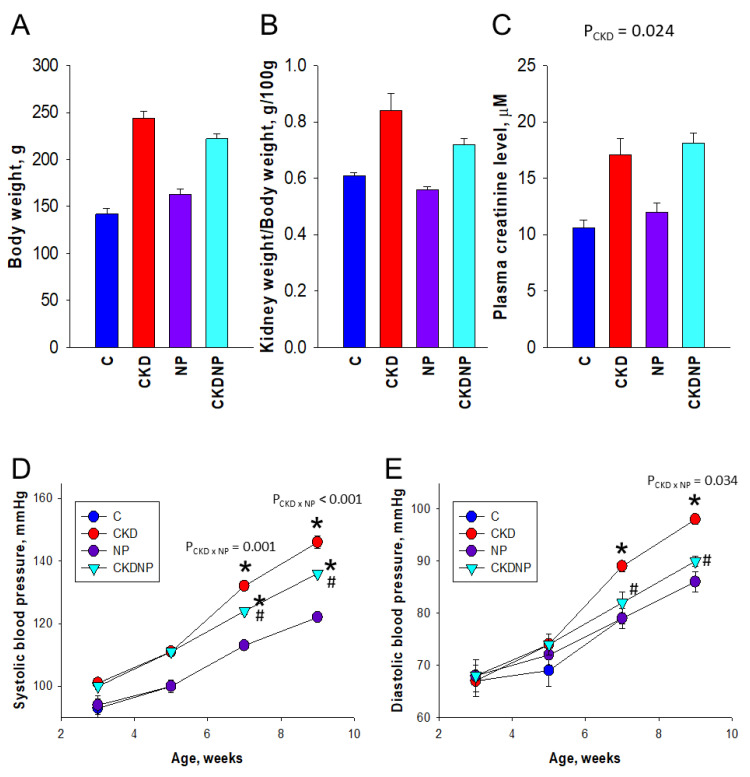
Effects of STS-loaded NPs on (**A**) body weight, (**B**) left kidney weight to body weight, (**C**) plasma creatinine level at week 9. (**D**) Systolic blood pressure and (**E**) diastolic blood pressure from week 3 to week 9. *n* = 8/group. A two-way ANOVA was performed for statistical analysis. P_CKD×NP_, interaction of CKD × NP; * *p* < 0.05 vs. C, # *p* < 0.05 vs. CKD.

Neither prenatal DEX exposure nor postnatal HF had an effect on body weight (BW) and the left kidney weight (KW)-to-BW ratio (Figure 3A,B). However, CKD resulted in an increase in plasma creatinine concentration (P_CKD_ = 0.024; Figure 3C). As shown in Figure 3D, baseline systolic BP values did not significantly differ among the groups. However, by seven and nine weeks, adenine-exposed juvenile rats exhibited elevated systolic BP. These increases in systolic BP in the CKD group were alleviated by STS-loaded NP treatment at both seven and nine weeks. A significant interaction between CKD and STS-loaded NP treatment was observed (P_CKD×NP_ = 0.001 at week 7 and <0.001 at week 9), indicating the efficacy of the treatment in reducing elevated BP. Notably, the changes in diastolic BP were more pronounced (Figure 3E). At nine weeks, STS-loaded NP treatment resulted in a reduction in systolic and diastolic BP by 10 mm Hg and 8 mm Hg, respectively.

### 3.3. H_2_S Pathway

**Figure 4:**
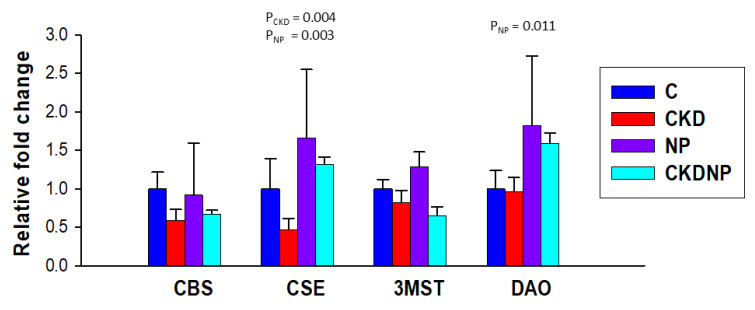
Renal mRNA expression of H_2_S-generating enzyme *CBS*, *CSE*, *3MST*, and *DAO*. *n* = 8/group; A two-way ANOVA was performed for statistical analysis.

To assess whether exogenous STS NPs influence the endogenous H_2_S pathway, we measured the mRNA expression of H_2_S-producing enzymes in the kidneys. As shown in Figure 4, CKD caused a significant reduction in renal *CSE* expression (P_CKD_ = 0.004), while STS-loaded NP treatment resulted in a significant increase in *CSE* expression (P_NP_ = 0.003). Neither CKD nor the STS-loaded NP treatment had a substantial effect on renal expression of *CBS* or *3MST*. However, STS-loaded NP treatment increased *DAO* expression (P_NP_ = 0.011).

### 3.4. NO Pathway

The plasma NO parameters are summarized in Table 2. Neither CKD nor the STS-loaded NP treatment had any effect on the plasma levels of L-citrulline, L-arginine, ADMA, or SDMA. However, CKD resulted in a significant reduction in the plasma L-arginine/ADMA ratio (P_CKD_ = 0.008). In contrast, the STS-loaded NP treatment significantly increased this ratio (P_NP_ = 0.029).

### 3.5. RAS

**Figure 5:**
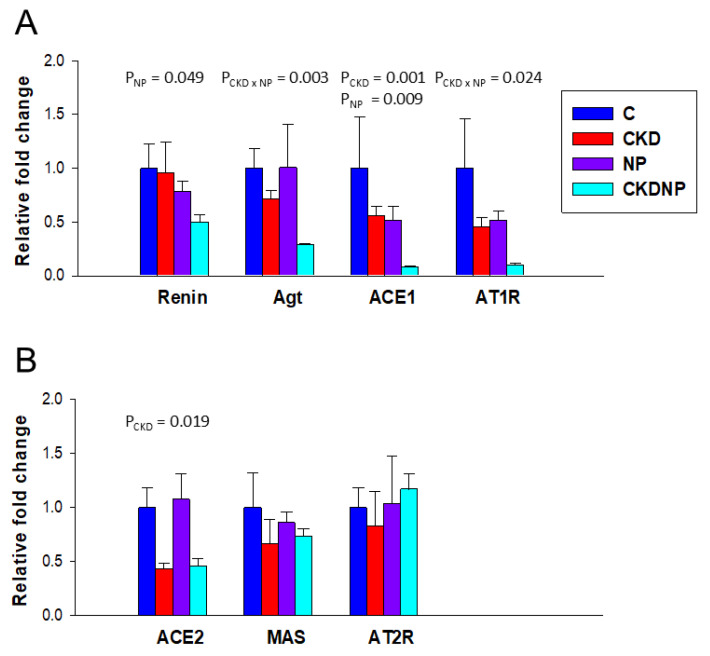
Renal mRNA expression of (**A**) the classical axis and (**B**) the nonclassical axis of the RAS. *n* = 8/group. A two-way ANOVA was performed for statistical analysis. P_CKD×NP_, interaction of CKD × NP.

To assess whether exogenous STS NPs influence the RAS to counteract the imbalance between vasoconstrictors and vasodilators, we measured RAS components in the kidneys (Figure 5). The RAS begins when renin cleaves angiotensinogen to form angiotensin I (Ang I). The classical RAS pathway converts Ang I to angiotensin II (Ang II) via *ACE1*, with Ang II binding to the *AT1R* to promote vasoconstriction. In contrast, the non-classical RAS, involving the *ACE2*-Ang-(1–7)-*MAS* and Ang II/Ang III-*AT2R* pathways, counterbalances the classical axis, exerting protective effects against vasoconstriction and hypertension.

Figure 5A shows that the STS-loaded NP treatment significantly inhibited the classical RAS axis, as indicated by a reduction in renal expression of renin (P_NP_ = 0.049) and *ACE1* (P_NP_ = 0.009). Although the STS-loaded NP treatment had no effect on renal *AGT* and *AT1R* expression, there was a synergistic effect of CKD and STS-loaded NP treatment in reducing their expression (P_CKD×NP_ = 0.003 and 0.024, respectively). Regarding the nonclassical axis (Figure 5B), CKD decreased renal *ACE2* expression in both the CKD and CKDNP groups (P_CKD_ = 0.019). However, neither CKD nor STS-loaded NP treatment affected *MAS* or *AT2R* expression.

## 4. Discussion

This study demonstrates that weekly STS-loaded NP treatment exerts a BP-lowering effect in juvenile CKD rats, reducing systolic BP by approximately 40%. Although the antihypertensive effect did not fully normalize BP, a reduction of 10 mm Hg in systolic BP is considered clinically significant. Our key contributions are summarized as follows: (1) we developed STS-loaded PEG-PLA-CPLA/F127 NPs that extend STS release for up to 7 days; (2) weekly STS-loaded NP treatment reduced systolic and diastolic BP by 10 mm Hg and 8 mm Hg, respectively, in juvenile CKD rats; (3) the protective effect of STS-loaded NPs is linked to increased renal expression of *CSE* and *DAO*; (4) CKD disrupts the NO signaling pathway, whereas STS-loaded NP therapy enhances NO bioavailability; and (5) the beneficial effects of STS-loaded NPs were associated with decreased levels of renin and *ACE1*.

While the antihypertensive effects of STS have been reported in kidney disease [5,7,17,18,19,20], our study extends this previous research by demonstrating that the PEG-PLA-CPLA/F127 NP system can sustain STS delivery. Weekly treatment with STS-loaded NPs effectively attenuates hypertension in juvenile CKD rats. Recent nanomedicine research has focused on developing H_2_S-triggered or therapeutic NP systems for H_2_S-activated sensing and therapy [9]. However, none of these systems has been used to deliver STS for the treatment of kidney disease and hypertension. To the best of our knowledge, we are the first to introduce the PEG-PLA-CPLA/F127 NP system for sustained STS delivery.

In clinical practice, STS is commonly administered intravenously due to its rapid degradation in the stomach [4]. In animal studies, STS has typically been delivered via intraperitoneal (i.p.) or intravenous (i.v.) routes [15,16,17]. Although oral administration of STS has shown antihypertensive effects in rats [5,7], the required therapeutic dose was five to ten times higher than that needed for the intravenous (i.v.) route, and its effects have not yet been evaluated in humans. In our NP delivery system, the STS dose was just one-tenth of the reported i.v. dose, suggesting a more efficient and targeted delivery method.

We have previously reported that administration of H_2_S precursors, such as N-acetylcysteine or L-cysteine, can protect against hypertension [21,22]. Since STS is also a precursor of H_2_S [23], our findings align with prior research supporting the therapeutic potential of exogenous H_2_S in hypertension. However, a major concern with exogenous H_2_S donors is the potential for toxicity, as they can lead to short-term increases in H_2_S to supraphysiological levels [24]. Our long-term, sustained NP delivery of STS may mitigate this concern, as it provides a controlled release of STS over time. This approach, with weekly administration, could be especially suitable for clinical translation in pediatric populations.

Our data indicate that treatment with STS-loaded NPs affects the H_2_S, NO, and RAS pathways. We observed that STS treatment increased the renal expression of both *CSE* and *DAO*, which are H_2_S-producing enzymes. *CSE* catalyzes the conversion of L-cysteine to H_2_S, while *DAO* contributes to H_2_S production through the metabolism of D-cysteine [3]. Prior research has shown that the D-cysteine pathway has an 80-fold greater H_2_S-generating activity than the L-cysteine pathway in the kidneys [25]. In previous studies, we found that young spontaneously hypertensive rats treated with high salt and supplemented with either L- or D-cysteine for 2 weeks were protected against hypertension and kidney damage at 12 weeks of age [19]. Whether the antihypertensive effect of STS-loaded NPs is due to a direct action or is also related to the regulation of the endogenous H_2_S-producing system requires further investigation.

Prior studies suggest that the BP-lowering effects of H_2_S may be related to enhanced NO signaling [1,26]. In our CKD model, the increase in BP induced by CKD was associated with a reduced L-arginine/ADMA ratio, a marker of NO bioavailability, which likely contributes to the development of hypertension. Treatment with STS-loaded NPs elevated this ratio. Therefore, the protective effect of STS-loaded NPs against hypertension may be attributed to the enhancement of the NO signaling pathway.

One potential beneficial effect of STS is its ability to rebalance the RAS [7]. Consistent with the use of RAS inhibitors, such as ACE inhibitors and angiotensin receptor blockers (ARBs), which are widely prescribed for the treatment of hypertension [27], our data demonstrate that inhibition of the classic RAS by STS-loaded NPs is associated with a BP-lowering effect. Reduction in the expression of renin, *AGT*, *ACE1*, and *AT1R* following STS-loaded NP therapy may help mitigate the detrimental effects of CKD-induced hypertension. However, we found that STS-loaded NPs had no effect on components of the nonclassical RAS axis, despite *ACE2* being inhibited by CKD.

This work has several limitations that should be considered. One limitation is that we only tested a single dose of STS-loaded NPs in the juvenile CKD rat model. Since the therapeutic effects of STS-loaded NPs can be influenced by various factors, such as morphology, surface modification, delivery routes, and kidney targeting efficiency, we selected an optimal condition for in vivo testing based on our data. Whether the BP-lowering effect of STS-loaded NPs is dose-dependent requires further investigation. Secondly, we did not assess potential sex differences in the response to STS-loaded NP treatment, as only male rats were used in this study due to their higher propensity for developing hypertension compared to females [28]. Lastly, while our findings indicate that STS has protective effects against hypertension, the scope of our study was limited to the evaluation of H_2_S, NO, and RAS systems. Given the multifaceted roles of H_2_S in kidney disease and hypertension [29,30], its interactions with other molecular pathways in protecting juvenile CKD rats against hypertension warrant further exploration. Further research is required in additional pediatric CKD models and human studies to fully evaluate the safety, efficacy, and translational potential of STS-loaded nanoparticles before they can be considered for clinical application.

## 5. Conclusions

In conclusion, our work not only develops an STS-loaded NP system for sustained delivery, but also demonstrates that STS treatment protects against hypertension in a juvenile CKD rat model. Our findings highlight the effects of STS-loaded NPs on key endogenous pathways, including H_2_S, NO, and the RAS, which may play a role in mitigating CKD-induced hypertension. The promising results of this study pave the way for the development of novel STS delivery NPs, with the potential to prevent hypertension in pediatric CKD, and offer a foundation for future clinical translation.

## Figures and Tables

**Table 1:** Primer sequences for qPCR analysis.

Gene	Gene Accession No	Sense	Anti-Sense
*CBS*	NM_012522.2	ATGCTGCAGAAAGGCTTCAT	GTGGAAACCAGTCGGTGTCT
*CSE*	NM_017074.2	CGCACAAATTGTCCACAAAC	GCTCTGTCCTTCTCAGGCAC
*3MST*	NM_138843.2	GGCTCAGTAAACATCCCATTC	TGTCCTTCACAGGGTCTTCC
*DAO*	NM_053626.1	CCCTTTCTGGAAAAGCACAG	CTCCTCTCACCACCTCTTCG
*Renin*	J02941.1	AACATTACCAGGGCAACTTTCACT	ACCCCCTTCATGGTGATCTG
*AGT*	XM_032887807.1	GCCCAGGTCGCGATGAT	TGTACAAGATGCTGAGTGAGGCAA
*ACE1*	U03734.1	CACCGGCAAGGTCTGCTT	CTTGGCATAGTTTCGTGAGGAA
*AT1R*	NM_030985.4	GCTGGGCAACGAGTTTGTCT	CAGTCCTTCAGCTGGATCTTCA
*ACE2*	NM_001012006.2	ACCCTTCTTACATCAGCCCTACTG	TGTCCAAAACCTACCCCACATAT
*MAS*	NM_203470.2	CATCTCTCCTCTCGGCTTTGTG	CCTCATCCGGAAGCAAAGG
*AT2R*	NM_012494.4	CAATCTGGCTGTGGCTGACTT	TGCACATCACAGGTCCAAAGA
*R18S*	X01117	GCCGCGGTAATTCCAGCTCCA	CCCGCCCGCTCCCAAGATC

**Table 2:** Plasma NO parameters.

Group	C	CKD	NP	CKDNP		*p*-Value	
					P_CKD_	P_NP_	P_CKD×NP_
L-citrulline (μM)	48.1 ± 8.4	54.8 ± 3.3	50.5 ± 4.6	59 ± 2.5	NS	NS	NS
L-arginine (μM)	266.5 ± 12	211.9 ± 8.9	260.2 ± 16.8	231.5 ± 12.2	NS	NS	NS
ADMA (μM)	2 ± 0.08	2.12 ± 0.08	1.84 ± 0.08	2.17 ± 0.14	NS	NS	NS
SDMA (μM)	1.41 ± 0.11	1.94 ± 0.08	1.64 ± 0.11	2.04 ± 0.1	NS	NS	NS
AAR (μM/μM)	134.7 ± 8.4	100.5 ± 4.1	144 ± 13.1	107.9 ± 4.6	0.008	0.029	NS

*n* = 8/group; ADMA = asymmetric dimethylarginine; SDMA = symmetric dimethylarginine; AAR = L-arginine/ADMA ratio. A two-way ANOVA was performed for statistical analysis. P_CKD×NP_, interaction of CKD × NP; NS = not significant.

## Data Availability

Data are contained within the article.

## References

[1] Kimura H. (2014). The physiological role of hydrogen sulfide and beyond. Nitric Oxide.

[2] Lobb I., Sonke E., Aboalsamh G., Sener A. (2015). Hydrogen sulphide and the kidney: Important roles in renal physiology and pathogenesis and treatment of kidney injury and disease. Nitric Oxide.

[3] Hsu C.N., Tain Y.L. (2018). Hydrogen Sulfide in Hypertension and Kidney Disease of Developmental Origins. Int. J. Mol. Sci..

[4] Zhang M.Y., Dugbartey G.J., Juriasingani S., Sener A. (2021). Hydrogen Sulfide Metabolite, Sodium Thiosulfate: Clinical Applications and Underlying Molecular Mechanisms. Int. J. Mol. Sci..

[5] Wyszynska T., Cichocka E., Wieteska-Klimczak A., Jobs K., Januszewicz P. (1992). A single pediatric center experience with 1025 children with hypertension. Acta Paediatr..

[6] Hadtstein C., Schaefer F. (2008). Hypertension in children with chronic kidney disease: Pathophysiology and management. Pediatr. Nephrol..

[7] Carey R.M., Sakhuja S., Calhoun D.A., Whelton P.K., Muntner P. (2019). Prevalence of Apparent Treatment-Resistant Hypertension in the United States. Hypertension.

[8] Nguyen I.T., Klooster A., Minnion M., Feelisch M., Verhaar M.C., van Goor H., Joles J.A. (2020). Sodium thiosulfate improves renal function and oxygenation in L-NNA–induced hypertension in rats. Kidney Int..

[9] Claramunt D., Gil-Peña H., Fuente R., García-López E., Loredo V., Frías O.H., Ordoñez F.A., Rodríguez J., Santos F. (2015). Chronic kidney disease induced by adenine: A suitable model of growth retardation in uremia. Am. J. Physiol. Physiol..

[10] Hsu C.N., Hou C.Y., Chang-Chien G.P., Lin S., Yang H.W., Tain Y.L. (2022). Sodium Thiosulfate Improves Hypertension in Rats with Adenine-Induced Chronic Kidney Disease. Antioxidants.

[11] Williams R.M., Jaimes E.A., Heller D.A. (2016). Nanomedicines for kidney diseases. Kidney Int..

[12] Chen W., Ni D., Rosenkrans Z.T., Cao T., Cai W. (2019). Smart H2S-Triggered/Therapeutic System (SHTS)-Based Nanomedicine. Adv. Sci..

[13] Chen C.K., Jones C.H., Mistriotis P., Yu Y., Ma X., Ravikrishnan A., Jiang M., Andreadis S.T., Pfeifer B.A., Cheng C. (2013). Poly (ethylene glycol)-block-cationic polylactide nanocomplexes of differing charge density for gene delivery. Biomaterials.

[14] Kabong M.A., Focke W.W., Du Toit E.L., Rolfes H., Ramjee S. (2020). Breakdown mechanisms of oil-in-water emulsions stabilised with Pluronic F127 and co-surfactants. Colloids Surf. A Physicochem. Eng. Asp..

[15] Williams R.M., Shah J., Tian H.S., Chen X., Geissmann F., Jaimes E.A., Heller D.A. (2018). Selective Nanoparticle Targeting of the Renal Tubules. Hypertension.

[16] Bode-Böger S.M., Scalera F., Ignarro L.J. (2007). The L-arginine paradox: Importance of the L-arginine/asymmetrical dimethylarginine ratio. Pharmacol. Ther..

[17] Snijder P.M., Frenay A.R., Koning A.M., Bachtler M., Pasch A., Kwakernaak A.J., van den Berg E., Bos E.M., Hillebrands J.L., Navis G. (2014). Sodium thiosulfate attenuates angiotensin II-induced hypertension, proteinuria and renal damage. Nitric Oxide.

[18] Kurian G.A., Mohan D., Balasubramanian E.D., Ravindran S. (2017). Renal mitochondria can withstand hypoxic/ischemic injury secondary to renal failure in uremic rats pretreated with sodium thiosulfate. Indian. J. Pharmacol..

[19] Baldev N., Sriram R., Prabu P., Gino A.K. (2015). Effect of mitochondrial potassium channel on the renal protection mediated by sodium thiosulfate against ethylene glycol induced nephrolithiasis in rat model. Int. Braz. J. Urol..

[20] Bijarnia R.K., Bachtler M., Chandak P.G., van Goor H., Pasch A. (2015). Sodium thiosulfate ameliorates oxidative stress and pre-serves renal function in hyperoxaluric rats. PLoS ONE.

[21] Tai I.H., Sheen J.M., Lin Y.J., Yu H.R., Tiao M.M., Chen C.C., Huang L.T., Tain Y.L. (2016). Maternal N-acetylcysteine therapy regulates hydrogen sulfide-generating pathway and prevents programmed hypertension in male offspring exposed to prenatal dexamethasone and postnatal high-fat diet. Nitric Oxide.

[22] Hsu C.N., Lin Y.J., Lu P.C., Tain Y.L. (2018). Early supplementation of D-cysteine or L-cysteine prevents hypertension and kidney damage in spontaneously hypertensive rats exposed to high-salt intake. Mol. Nutr. Food Res..

[23] Olson K.R., Deleon E.R., Gao Y., Hurley K., Sadauskas V., Batz C., Stoy G.F. (2013). Thiosulfate: A readily accessible source of hydrogen sulfide in oxygen sensing. Am. J. Physiol. Regul. Integr. Comp. Physiol..

[24] Li Z., Polhemus D.J., Lefer D.J. (2018). Evolution of Hydrogen Sulfide Therapeutics to Treat Cardiovascular Disease. Circ. Res..

[25] Shibuya N., Kimura H. (2013). Production of hydrogen sulfide from D-cysteine and its therapeutic potential. Front. Endocrinol..

[26] Wu D., Hu Q., Zhu D. (2018). An Update on Hydrogen Sulfide and Nitric Oxide Interactions in the Cardiovascular System. Oxidative Med. Cell. Longev..

[27] Chen Y.J., Li L.J., Tang W.L., Song J.Y., Qiu R., Li Q., Xue H., Wright J.M. (2018). First-line drugs inhibiting the renin angiotensin system versus other first-line antihypertensive drug classes for hypertension. Cochrane Database Syst. Rev..

[28] Reckelhoff J.F. (2001). Gender differences in the regulation of blood pressure. Hypertension.

[29] Kasinath B.S., Feliers D., Lee H.J. (2018). Hydrogen sulfide as a regulatory factor in kidney health and disease. Biochem. Pharmacol..

[30] Peleli M., Zampas P., Papapetropoulos A. (2022). Hydrogen Sulfide and the Kidney: Physiological Roles, Contribution to Pathophysiology, and Therapeutic Potential. Antioxid. Redox Signal..

